# Multi-Variable Transformer-Based Meta-Learning for Few-Shot Fault Diagnosis of Large-Scale Systems

**DOI:** 10.3390/s25092941

**Published:** 2025-05-07

**Authors:** Weiyang Li, Yixin Nie, Fan Yang

**Affiliations:** Department of Automation, Tsinghua University, Beijing 100084, China; liwy18@mails.tsinghua.edu.cn (W.L.); nieyx22@mails.tsinghua.edu.cn (Y.N.)

**Keywords:** fault diagnosis, few-shot learning, time series data, meta-learning, transformer

## Abstract

Fault diagnosis in large-scale systems presents significant challenges due to the complexity and high dimensionality of data, as well as the scarcity of labeled fault data, which are hard to obtain during the practical operation process. This paper proposes a novel approach, called Multi-Variable Meta-Transformer (MVMT), to tackle these challenges. In order to deal with the multi-variable time series data, we modify the Transformer model, which is the currently most popular model on feature extraction of time series. To enable the Transformer model to simultaneously receive continuous and state inputs, we introduced feature layers before the encoder to better integrate the characteristics of both continuous and state variables. Then, we adopt the modified model as the base model for meta-learning—more specifically, the Model-Agnostic Meta-Learning (MAML) strategy. The proposed method leverages the power of Transformers for handling multi-variable time series data and employs meta-learning to enable few-shot learning capabilities. The case studies conducted on the Tennessee Eastman Process database and a Power-Supply System database demonstrate the exceptional performance of fault diagnosis in few-shot scenarios, whether based on continuous-only data or a combination of continuous and state variables.

## 1. Introduction

Large-scale systems, such as industrial machinery, power grids, and transportation networks, are fundamental to modern infrastructure. Ensuring their dependability and safety requires precise and timely fault detection and diagnosis (FDD). Traditional knowledge-driven fault diagnosis methods rely heavily on extensive mechanistic knowledge and expert experience. However, as system complexity increases, constructing a diagnostic model solely based on first principles becomes infeasible. Consequently, since the early 21st century, FDD in large-scale systems has transitioned into the data-driven era.

With the continuous advancement of deep learning, its application in FDD has garnered significant attention. Deep neural networks excel at extracting features, allowing for precise modeling of complex nonlinear relationships in large-scale systems. However, as network size grows, so does the demand for vast amounts of labeled data. One of the key challenges in data-driven FDD is the limited availability of fault samples for each specific fault category. To address this issue, meta-learning has emerged as a promising approach. Meta-learning, or “learning to learn” [1], enables models to generalize from a few examples, making it particularly suitable for fault diagnosis in large-scale systems where labeled data are scarce. It involves deriving prior knowledge from numerous similar few-shot fault diagnosis tasks and utilizing this prior knowledge to build a model that quickly adapts to new fault scenarios. Depending on the type of prior knowledge, meta-learning can be classified into initialization-based, metric-based, and optimizer-based methods.

Model-Agnostic Meta-Learning (MAML) is one of the most effective initialization-based approaches. MAML learns an optimal set of initial network weights from a large number of source domain samples, which serves as prior knowledge [2]. This knowledge is then applied to the target domain, enabling rapid adaptation for few-shot fault diagnosis tasks. Initially, MAML was implemented using Convolutional Neural Networks (CNNs), which are well suited for feature extraction from 2D image data. However, when analyzing multivariate time series data in complex systems, CNNs may fall short due to their limited ability to capture temporal dependencies. Therefore, introducing Recurrent Neural Networks (RNNs) or Long Short-Term Memory (LSTM) networks into the MAML strategy as base models seems to be a valuable solution for time series analysis challenges. But the recurrent nature of these architectures requires extensive computations and backpropagation, significantly slowing down the meta-training process. As a result, traditional recurrent architectures, despite their effectiveness in multivariate time series classification, are not well suited for MAML-based FDD.

To overcome these limitations, recent advancements in deep learning have introduced Transformers, which have demonstrated exceptional performance in handling complex, high-dimensional data. Unlike RNNs, Transformers utilize position encoding to retain sequence information without relying on recurrence, making them a compelling choice for meta-learning applications. In real-world fault diagnosis scenarios, multivariate time series data often consist of both continuous analog and discrete state variables. To effectively capture the unique characteristics of these variable types, we propose an enhanced Transformer model. In our approach, analog and state variables are processed through separate embedding layers to extract their respective feature representations before being fed into the Transformer’s encoder for Multi-Head Self-Attention operations. This architecture ensures that both types of variables are effectively merged and utilized for fault diagnosis.

By integrating this enhanced multi-variable Transformer model with MAML, we introduce the Multi-Variable Meta-Transformer (MVMT), a novel approach specifically designed for small-sample fault diagnosis in large-scale systems. Unlike existing methods, MVMT effectively captures the unique characteristics of both continuous analog and discrete state variables through separate embedding layers, ensuring a more comprehensive representation of multivariate time series data. Furthermore, by leveraging the self-attention mechanism of Transformers, our approach overcomes the limitations of recurrent structures, significantly improving both computational efficiency and adaptation speed in meta-learning.

To rigorously evaluate the effectiveness of MVMT, we conducted extensive experiments on the Tennessee Eastman Process (TEP) dataset and a satellite Power-Supply System dataset. Comparative analysis with state-of-the-art neural networks, including LSTM, CNN, and Vision Transformer (ViT) combined with meta-learning, demonstrates that MVMT achieves superior fault diagnosis accuracy while maintaining high training efficiency. This work makes three key contributions: (1) introducing a Transformer-based meta-learning framework tailored for multivariate time series fault diagnosis in the few-shot scenario, (2) proposing a novel embedding mechanism that effectively integrates both analog and state variables, and (3) validating the effectiveness of MVMT through comprehensive experimental comparisons on benchmark and real-world datasets. Our findings underscore the potential of Transformer-based meta-learning in addressing the challenges of few-shot fault diagnosis in complex large-scale systems.

The article will progress as follows: Section 2 will discuss related work, focusing on the use of Transformers in fault diagnosis and advancements in meta-learning for addressing fault diagnosis with limited data. Section 3, Methodology, will detail the MVMT method, outlining its model structure and training approaches. Section 4 will cover the experimental setup, detailing the datasets used and the experimental configurations. Section 5 will showcase the experimental results and offer a detailed analysis. Finally, Section 6 will present conclusions regarding the MVMT method.

## 2. Related Work

Fault diagnosis in large-scale systems has evolved significantly over the years, with recent advancements in deep learning, particularly Transformers, and meta-learning techniques addressing key challenges such as high-dimensional data and limited labeled examples.

### 2.1. Transformers in Fault Diagnosis

The self-attention deep network Transformer was originally proposed for natural language-processing tasks [3]. Due to its self-attention mechanism, Transformer has demonstrated excellent capabilities in handling sequential data. This mechanism enables Transformer to capture long-term temporal dependencies and complex features, making it intuitively well suited for time series data analysis. In the context of fault diagnosis, Transformer has shown great potential in extracting features from long-term sequential data.

**Figure 1:**
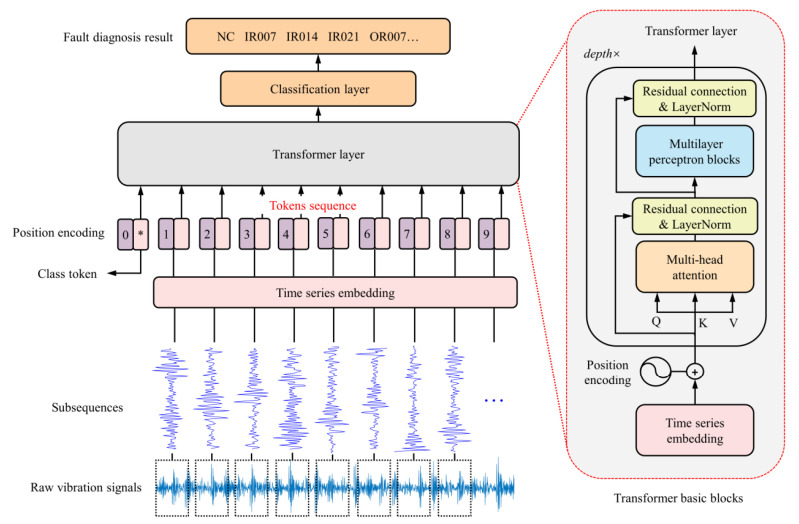
Schematic diagram of the Time Series Transformer (TST) structure [4]. * is an additional token for classification.

In recent years, Transformer models have demonstrated significant potential in the field of mechanical equipment fault diagnosis. Jin et al. [4] proposed a rotating machinery fault diagnosis method based on the time series Transformer (TST). This method introduced a new time series tokenizer to convert one-dimensional data into a format suitable for Transformer processing. Experimental results showed that TST achieved higher fault diagnosis accuracy than traditional CNN and RNN models on the CWRU, XJTU-SY, and UCONN datasets. As the first attempt to apply Transformer to the field of mechanical fault diagnosis, it directly tokenizes the time series data, which is an operation that converts time series data into discrete tokens, and then feeds them into the classical Transformer model. The overall structure is shown in Figure 1.

Hou et al. [5] designed a bearing fault diagnosis method based on Transformer and ResNet (TAR). This method first used a one-dimensional convolutional layer to perform feature separation and embedding on the original signal and then passed it to the Transformer encoder and ResNet framework for feature extraction. The application of transfer learning strategies reduced the training difficulty of new tasks. Experimental results on the CWRU dataset showed that TAR achieved a fault diagnosis accuracy of 99.90% under noise-free conditions, and its average fault diagnosis accuracy under different signal-to-noise ratios was also higher than that of comparative methods.

Fang et al. [6] proposed a lightweight Transformer called CLFormer for rotating machinery fault diagnosis. This model replaced the original embedding module with a convolutional embedding module and used a linear self-attention mechanism to reduce model complexity. In experiments, the number of parameters of CLFormer was reduced from 35.22K in the original Transformer to 4.88K, and the fault diagnosis accuracy was improved from 82.68% to 90.53%, demonstrating its practical value.

Hou et al. [7] designed a multi-feature parallel fusion rolling bearing fault diagnosis method based on Transformer, called Diagnosisformer. This method first performed fast Fourier transform on the original vibration data to extract frequency domain features and then extracted local and global features of the data through a multi-feature parallel fusion encoder. Experimental results on self-made rotating machinery fault diagnosis data and the CWRU dataset showed that the average diagnosis accuracy of Diagnosisformer reached 99.84% and 99.85%, respectively, significantly outperforming methods such as CNN, CNN-LSTM, RNN, LSTM, and GRU.

Zhang et al. [8] developed the Time Series Vision Transformer (TSViT), integrating a convolutional layer to extract local features and a Transformer encoder to capture long-term temporal patterns. TSViT demonstrated exceptional diagnostic capabilities, achieving 100% average accuracy on two test sets and 99.99% on another. Nascimento et al. [9] introduced T4PdM, a deep neural network based on the Transformer architecture for fault diagnosis of rotating machinery. Their model achieved overall accuracies of 99.98% and 98% on the MaFaulDa and CWRU datasets, respectively, showcasing its effectiveness in identifying multiple types of faults.

Yang et al. [10] proposed a signal Transformer (SiT) based on an attention mechanism and applied it to bearing fault diagnosis research. This method used short-time Fourier transform to convert one-dimensional fault signals into two-dimensional images, which were then fed into the Transformer model for classification. Experimental results showed that, compared with traditional convolutional models, SiT performed better in processing fault time information and improved classification accuracy.

In recent years, Transformer-based fault diagnosis methods have advanced rapidly. Starting from the initial TST model, a series of new methods and technologies have emerged and evolved at a fast pace. However, despite these advancements, the application of Transformer models in fault diagnosis still faces several key limitations:

- Data Scarcity: Although Transformer models excel on large-scale datasets, acquiring sufficient labeled data remains a major challenge in complex system fault diagnosis, particularly in few-shot learning scenarios. In many industrial applications, system failures are rare, leading to a severe shortage of samples for each fault type. Despite its powerful feature extraction capabilities, Transformer typically relies on a vast amount of labeled data for effective training, which is often impractical in real-world settings. While some unsupervised learning methods have been proposed, they still require a large number of unlabeled samples and fail to fundamentally overcome the limitations imposed by sample scarcity on model performance.
- Multivariate Issues: As observed in previous studies, nearly all research relies on publicly available bearing datasets to benchmark against state-of-the-art methods. While this approach intuitively evaluates the performance of proposed methods, bearing datasets inherently consist of single-variable time series data collected from vibration sensors. In contrast, data in complex systems often comprise multivariate time series, making it challenging for bearing datasets to accurately reflect the effectiveness of Transformer-based fault diagnosis methods in more diverse and realistic application scenarios.
- Time Series Nature: Since bearing datasets typically consist of high-frequency sampled time series data, researchers often tokenize or transform them into the frequency domain for processing. However, in large and complex systems—such as process industries and spacecraft—the sampling frequency is usually much lower. Due to limited sampling rates and fewer data points, conventional operations like segmentation or tokenization may fail to effectively extract meaningful features. Consequently, determining how to process these measurement variables and efficiently input them into Transformer models remains an urgent challenge that needs to be addressed.
- State Variables: Similar to the previous issues, bearing datasets primarily collect vibration signals, which are analog data. However, in complex systems, measurement data often include crucial state variables, such as switch states and operating modes, which significantly impact system behavior. Despite their importance, these state variables have not been adequately considered in model inputs. Effectively processing these state variables and seamlessly integrating their features with analog data remains a critical challenge in fault diagnosis.

### 2.2. Advancements of Meta-Learning in Fault Diagnosis

In recent years, meta-learning has emerged as a promising approach to address the challenges of fault diagnosis in complex systems, particularly under conditions of limited data availability and varying operational environments. Previously, several model-driven methods were proposed to tackle these challenges—for example, model-driven fault diagnosis approaches for subsea blowout preventer systems [11,12]. However, data-driven deep learning methods typically require large amounts of labeled data and often struggle to generalize across different working conditions, especially in complex closed-loop control systems.

Meta-learning, or “learning to learn”, aims to enable models to rapidly adapt to new tasks with minimal data by leveraging prior experience from related tasks. Model-Agnostic Meta-Learning (MAML) is a prominent meta-learning approach that has been widely adopted due to its ability to quickly adapt to new tasks with only a few examples. MAML optimizes the model’s initial parameters, enabling rapid fine-tuning for specific tasks, making it particularly well suited for fault diagnosis scenarios where labeled fault data are scarce.

One notable application of meta-learning in fault diagnosis is the development of the Meta-Learning Fault Diagnosis (MLFD) framework. This approach utilizes MAML to learn initial model parameters that can be quickly fine-tuned to new fault diagnosis tasks with limited data. By converting raw vibration signals into time–frequency images and organizing tasks according to an *N*-way *K*-shot protocol, MLFD has demonstrated superior performance in few-shot fault diagnosis scenarios [13]. It has shown outstanding performance in bearing fault diagnosis tasks under complex and variable operating conditions. Meanwhile, Chang and Lin [14] introduced an adaptive learning rate and proposed the MLALR method to further enhance fault diagnosis accuracy.

To further enhance adaptability, researchers have combined meta-learning with Neural Architecture Search (NAS). The MetaNAS approach employs meta-learning to find optimal initial parameters, allowing the model to identify the most effective network architecture for new fault modes with only a few gradient updates. This integration addresses the challenge of diagnosing novel fault types under small-sample conditions [15].

Another advancement is the Meta-GENE framework, which focuses on domain generalization in intelligent fault diagnosis. By incorporating gradient aligning and semantic matching strategies within a meta-learning framework, Meta-GENE enhances the model’s ability to generalize across different domains, making it particularly useful in industrial environments with diverse operating conditions [16].

Therefore, there has been a growing interest in combining meta-learning with Transformers to leverage the strengths of both approaches. Transformers, with their powerful feature extraction capabilities, serve as an excellent base model for meta-learning. The integration of Transformers with MAML allows for effective handling of multi-variable time series data and enables fast adaptation to new fault types with limited labeled data. This combination has demonstrated superior performance in few-shot fault diagnosis tasks, offering a robust and scalable solution for large-scale systems.

In summary, the application of Transformers in fault diagnosis and the advancements in meta-learning techniques have significantly contributed to addressing the challenges of high-dimensional data and limited labeled examples. The integration of these approaches, as proposed in this work, holds great potential for improving the accuracy, efficiency, and adaptability of fault diagnosis models in complex, large-scale systems.

## 3. Methodology

### 3.1. Overall Framework of the Multi-Variable Meta-Transformer (MVMT)

**Figure 2:**
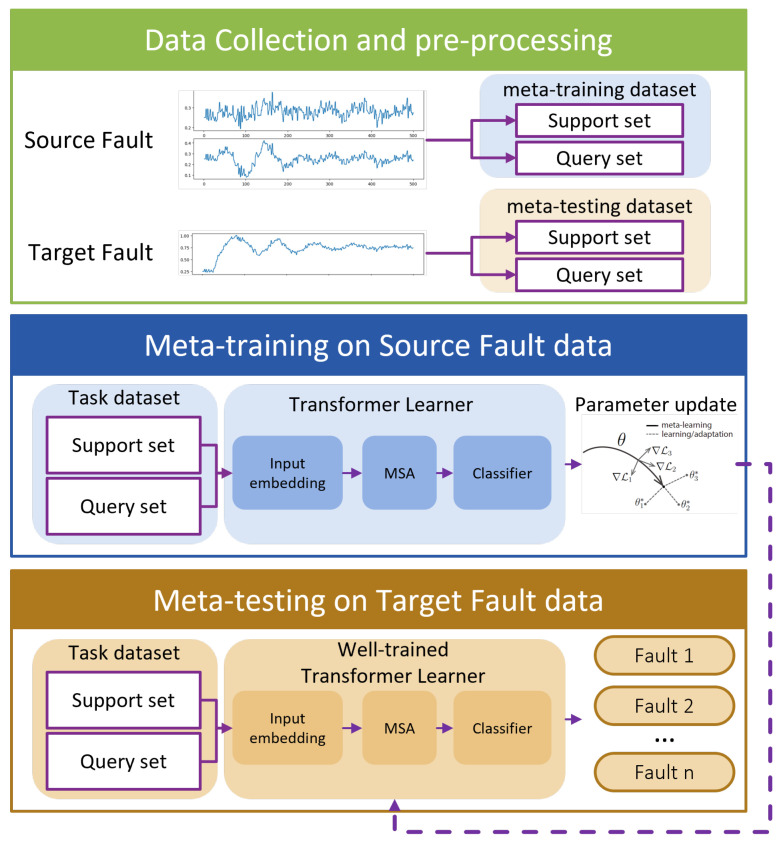
Overall framework of the Multi-Variable Meta-Transformer.

By seamlessly integrating MAML, the Transformer model, and multi-variable fusion strategies, we introduce the Multi-Variable Meta-Transformer (MVMT). Designed for high efficiency and accuracy, MVMT enables rapid feature extraction and classification, even when only a few samples of both analog and state variables are available. The overall framework of MVMT is depicted in Figure 2.

The MVMT framework is structured into three key stages:

- Data Collection and Pre-processing: Faults are classified as either source faults or target faults based on the number of available samples. Source faults, being common, have a sufficient number of samples, whereas target faults are rare and have only a limited number of samples. These faults are then allocated to a meta-training task set and a meta-testing task set, respectively. Notably, the faults in the meta-testing set are entirely distinct from those in the meta-training set. Unlike conventional train–test splits, fault types included in the meta-training set do not reappear in the meta-testing set.
- Meta-Training Phase: In this stage, a MAML strategy is employed to train the multi-variable Transformer model, optimizing its initial parameters. This process yields a well-initialized multi-variable Transformer encoder, which serves as the foundation for the subsequent meta-testing phase.
- Meta-Testing Phase: Here, the pre-trained multi-variable Transformer model is fine-tuned using the limited samples of rare faults, enabling it to effectively perform the final few-shot fault diagnosis task.

The following sections provide a detailed explanation of the meta-learning techniques, the Transformer model, and the overall architecture and training process of the MVMT model.

### 3.2. Meta-Learning Framework

To enhance the few-shot learning capability of the base model, we integrate it into a meta-learning framework, specifically leveraging Model-Agnostic Meta-Learning (MAML). MAML optimizes model parameters to facilitate rapid adaptation to new tasks, even with only a few available data samples.

In the context of few-shot learning for fault diagnosis, each task *T* consists of *N* fault types (*N*-way), with *K* support samples (*K*-shot) per type, *Q* query samples, and a specific loss function $L_{T_{i}}$.

The core idea of MAML is to determine an optimal model initialization θ such that, under the distribution of the entire classification task set, a model initialized with θ can be quickly trained to achieve high performance. In other words, this initialization enables the model to efficiently adapt to various classification tasks within the task set.

The MAML algorithm consists of two key optimization processes: meta-training and meta-testing. During meta-training, the model learns from multiple tasks to enhance its ability to adapt rapidly. The objective is to identify an optimal initial parameter θ from the source domain dataset during meta-training and transfer it as prior knowledge to the target domain. This allows the model to fine-tune efficiently with only a few gradient updates in the target domain, enabling effective adaptation to new tasks.

**Figure 3:**
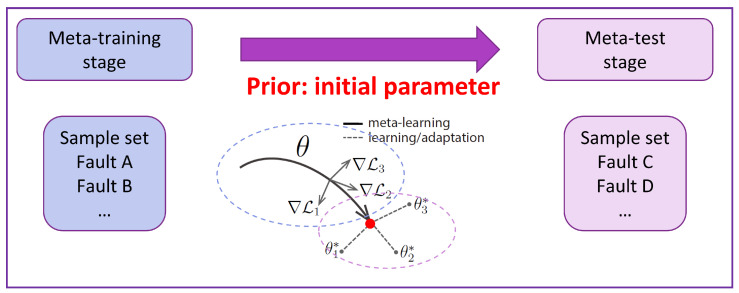
Relationship between meta-training and meta-testing. The red dot denotes the optimal initial parameters (prior) learned during the meta-training phase.

Figure 3 illustrates the relationship between the meta-training and meta-testing phases.

The overall process of the MAML algorithm is as follows:

1. Sample *B* tasks from the task distribution $T_{i}∼p\left(T\right)$. Each task consists of *N* fault types, with *K* support samples for task-specific training and *Q* query samples for task-specific validation.
2. For each task $T_{i}$, use the K×N support samples to compute the updated parameters $\theta_{i}^{\prime }$ through *S* iterations of inner-loop training:
  (1) $$\theta_{i}^{\prime }=\theta -\alpha \nabla_{\theta }L_{T_{i}}\left(f_{\theta }\right)$$
  where α is the inner-loop learning rate.
3. Evaluate the updated model using the query sets from all *B* tasks. The task-specific loss function $L_{T_{i}}$ is used to compute the overall meta-objective:
  (2) $$\min_{\theta }\sum_{T_{i}∼p\left(T\right)}L_{T_{i}}\left(f_{\theta_{i}^{S}}\right)$$
4. Update the model parameters θ using the meta-gradient:
  (3) $$\theta \leftarrow \theta -\beta \nabla_{\theta }\sum_{T_{i}∼p\left(T\right)}L_{T_{i}}\left(f_{\theta_{i}^{S}}\right)$$
  where β is the meta-learning rate (outer-loop learning rate).

**Figure 4:**
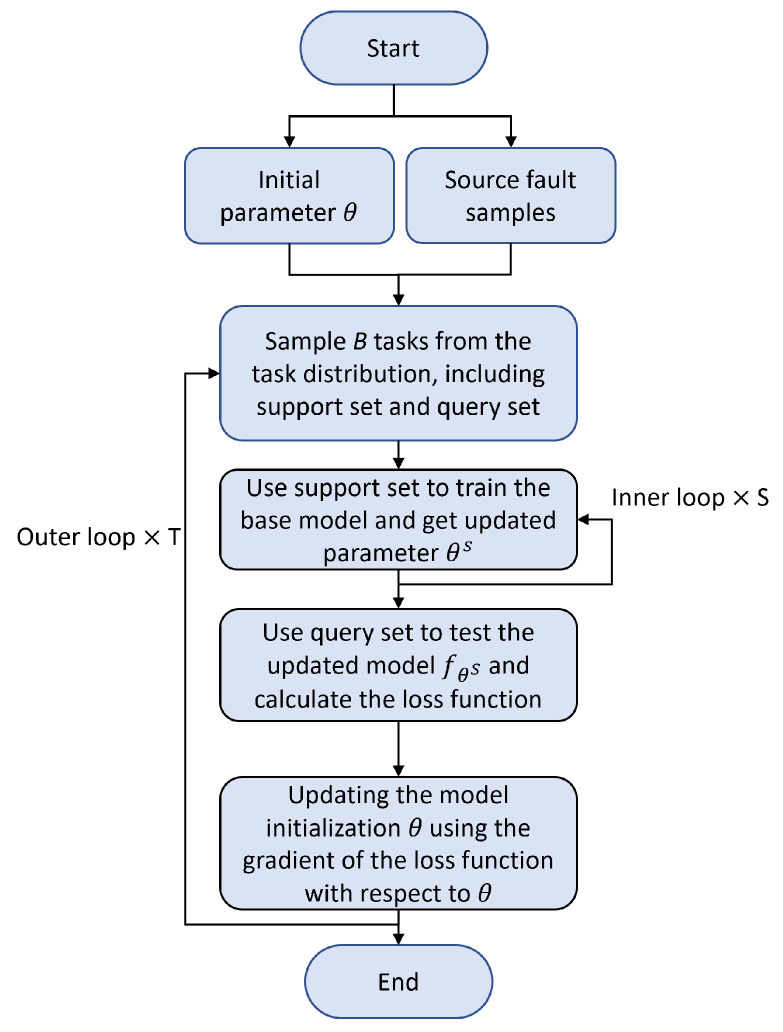
MAML algorithm workflow.

Figure 4 illustrates the workflow of the MAML algorithm. Step 2 represents the inner loop of the process, while steps 1 through 4 form the outer loop.

The objective of the outer loop is to optimize the model’s initial parameters through training on multiple tasks. Specifically, the outer loop attempts to find a good initialization parameter θ that allows the model to quickly adapt to new tasks. The outer loop updates the initialization parameters using the meta-loss from multiple tasks, thereby enhancing the model’s generalization ability.

The inner loop is used for short-term optimization on each task, with the goal of adjusting the model parameters through a few gradient updates based on the task’s data. The optimization process in the inner loop typically uses gradient descent to update the model parameters. For each task $T_{i}$, the model is fine-tuned on the task-specific training data during the inner loop.

While the objectives of the outer and inner loops are different, they are interdependent. The inner loop fine-tunes the parameters on each task, enabling the model to rapidly adapt to the task-specific features. Meanwhile, the outer loop optimizes the initial parameters based on the training results from multiple tasks, allowing the initialization to generalize to different tasks. In MAML, the outer and inner loops alternate, progressively enhancing the model’s adaptability through multiple training iterations.

For more details, please check Algorithm 1.

**Algorithm 1** MAML for Classification**Input:**        
α: learning rate for inner updates           β: learning rate for meta-update           p(T): distribution over tasks**Output:**     Model parameters θ**1:**          Initialize model parameters θ **2:**          **while** not done **do** **3:**             Sample batch of tasks $T_{i}∼p\left(T\right)$
**4:**             **for all** $T_{i}$ **do** **5:**                Sample *K* datapoints $D_{T_{i}}=\left\{\left(x_{k},y_{k}\right)\right\}$ **6:**                Evaluate $\nabla_{\theta }L_{T_{i}}\left(f_{\theta }\right)$ using $D_{T_{i}}$ **7:**                Compute adapted parameters with gradient descent:                $\theta_{i}^{\prime }=\theta -\alpha \nabla_{\theta }L_{T_{i}}\left(f_{\theta }\right)$ **8:**             **end for**
**9:**             Sample new datapoints $D_{T_{i}}^{\prime }=\left\{\left(x_{k},y_{k}\right)\right\}$ **10:**           Compute meta-objective:             $\theta \leftarrow \theta -\beta \nabla_{\theta }\sum_{T_{i}∼p\left(T\right)}L_{T_{i}}\left(f_{\theta_{i}^{\prime }}\right)$ **11:**        **end while**


During meta-testing, the model uses the learned initial parameters to quickly adapt to new tasks with a few gradient steps, enabling effective few-shot learning.

Meta-SGD is an extension of MAML that not only learns the initial parameters but also learns the learning rates for each parameter during the meta-training process. This provides an additional layer of flexibility and can lead to faster convergence and better performance.

The main modification in Meta-SGD is that each parameter $\theta_{i}$ has an associated learning rate $\alpha_{i}$. During the meta-training phase, both the initial parameters and the learning rates are updated.

During meta-testing, the model uses the learned initial parameters and learning rates to quickly adapt to new tasks with a few gradient steps, enhancing the effectiveness of few-shot learning. For more details, please check Algorithm 2.

In practical fault diagnosis applications, the number of fault categories in tasks from the source domain may not necessarily match those in the target domain. To address this issue, we introduced an adaptive classifier. While traditional MAML uses a fixed classification head, the adaptive classifier generates a specific classifier for each task, enabling dynamic adaptation to the number of categories in different tasks. For more details, please check Algorithm 3.
**Algorithm 2** Meta-SGD for Classification**Input:**       α: learning rates for inner updates (learned)          β: learning rate for meta-update          p(T): distribution over tasks**Output:**      Model parameters θ and learning rates α**1:**         Initialize model parameters θ and learning rates α **2:**         **while** not done **do**
**3:**
           Sample batch of tasks $T_{i}∼p\left(T\right)$ **4:**            **for all** $T_{i}$ **do** **5:**               Sample *K* datapoints $D_{T_{i}}=\left\{\left(x_{k},y_{k}\right)\right\}$ **6:**               Evaluate $\nabla_{\theta }L_{T_{i}}\left(f_{\theta }\right)$ using $D_{T_{i}}$ **7:**               Compute adapted parameters with learned                  gradient descent:                  $\theta_{i}^{\prime }=\theta -\alpha ⊙\nabla_{\theta }L_{T_{i}}\left(f_{\theta }\right)$ **8:**            **end for** **9:**
           Sample new datapoints $D_{T_{i}}^{\prime }=\left\{\left(x_{k},y_{k}\right)\right\}$ **10:**             Compute meta-objective:               $\theta \leftarrow \theta -\beta \nabla_{\theta }\sum_{T_{i}∼p\left(T\right)}L_{T_{i}}\left(f_{\theta_{i}^{\prime }}\right)$               $\alpha \leftarrow \alpha -\beta \nabla_{\alpha }\sum_{T_{i}∼p\left(T\right)}L_{T_{i}}\left(f_{\theta_{i}^{\prime }}\right)$ **11:**       **end while**

**Algorithm 3** MAML with adaptive classifier.**Input**         α: Inner-loop learning rate           β: Meta-learning rate           p(T): Task distribution**Output**     Optimized model parameters θ**1:**          Initialize model parameters θ **2:**          **while** not converged **do** **3:**              Sample a batch of tasks $T_{i}$ from task distribution p(T) **4:**              **for each** task $T_{i}$ **do** **5:**                  Sample support set $D_{T_{i}}^{\mathrm{support}}$ and query set $D_{T_{i}}^{\mathrm{query}}$ **6:**                  Construct the adaptive classifier $g_{\phi }$ using $D_{T_{i}}^{\mathrm{support}}$ **7:**                  Compute task-specific loss:                   $L_{T_{i}}=\sum_{\left(x_{j},y_{j}\right)\in D_{T_{i}}^{\mathrm{support}}}\ell \left(g_{\phi }\left(f_{\theta }\left(x_{j}\right)\right),y_{j}\right)$ **8:**                  Compute task-specific parameter updates:                     $\theta_{T_{i}}^{\prime }=\theta -\alpha \nabla_{\theta }L_{T_{i}}$ **9:**              **end for** **10:**            Compute meta-loss on the query set:              $L_{\mathrm{meta}}=\sum_{T_{i}}\sum_{\left(x_{j},y_{j}\right)\in D_{T_{i}}^{\mathrm{query}}}\ell \left(g_{\phi }\left(f_{\theta_{T_{i}}^{\prime }}\left(x_{j}\right)\right),y_{j}\right)$ **11:**            Update meta-parameters:              $\theta \leftarrow \theta -\beta \nabla_{\theta }L_{\mathrm{meta}}$ **12:**       **end while**


### 3.3. Multi-Variable Transformer

The Transformer [3], a deep network based on self-attention, was initially proposed for natural language-processing tasks and has demonstrated exceptional capability in capturing long-range dependencies. In this study, we extend the Transformer model to multi-variable time series data, which are prevalent in complex systems such as process industries, energy systems, and on-orbit spacecraft.

#### 3.3.1. Overall Structure of Transformer

**Figure 5:**
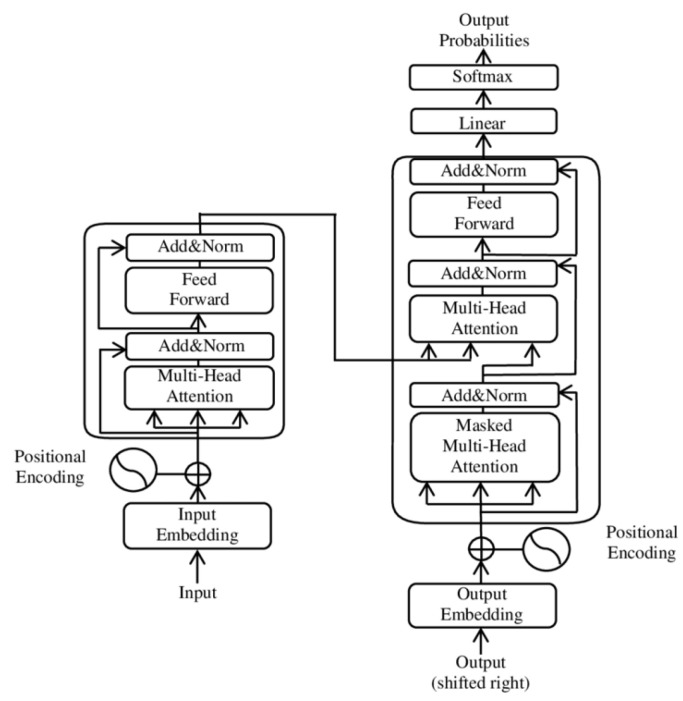
Transformer structure includes multiple encoders and decoders [17].

The overall architecture of the Transformer, as shown in Figure 5, consists of two main components: the encoder and the decoder. The encoder extracts high-level features from the input data, while the decoder utilizes these features to generate predictions.

The encoder consists of *N* identical layers, each comprising two key components:

1. Multi-Head Self-Attention Mechanism
2. Feed-Forward Network (FFN)

Given an input time series $X=(x_{1},x_{2},\ldots ,x_{T})$, it is first processed through an embedding layer and positional encoding before being fed into the Transformer encoder:

(4) $$H^{\left(0\right)}=\mathrm{Embedding}\left(X\right)+\mathrm{PE}\left(X\right)$$

The encoder computes representations as follows:

(5) $$H^{\left(l\right)}=\mathrm{FFN}\left(\mathrm{MultiHead}\left(H^{\left(l-1\right)},H^{\left(l-1\right)},H^{\left(l-1\right)}\right)\right)$$

where

- $H^{\left(l\right)}$ is the representation at layer *l*.
- Multi-head attention allows the model to learn various time dependencies.
- The Feed-Forward Network (FFN) applies nonlinear transformations to enhance feature representations.

The decoder has a similar structure but includes an additional cross-attention layer to incorporate information from the encoder output. The decoder computation is as follows:

(6) $$Z^{\left(l\right)}=\mathrm{FFN}\left(\mathrm{MultiHead}\left(Z^{\left(l-1\right)},H,H\right)\right)$$

where

- $Z^{\left(l\right)}$ is the decoder representation at layer *l*.
- The cross-attention mechanism allows the decoder to access encoder representations H, improving prediction performance.

The decoder’s final output is passed through a linear layer followed by a Softmax function to obtain the final prediction:

(7) $${\hat{y}}_{t}=\mathrm{Softmax}\left(W_{o}Z^{\left(N\right)}+b_{o}\right)$$

where

- $W_{o}$ and $b_{o}$ are learnable parameters.
- For classification tasks, Softmax outputs a probability distribution over classes.
- For regression tasks, the model directly outputs the predicted values.

Multi-Head Self-Attention is a crucial component of the Transformer model, enabling it to dynamically assign varying levels of importance to different variables. The self-attention mechanism is formulated as follows:

#### 3.3.2. Self-Attention Mechanism

The core of the Transformer model is the self-attention mechanism, which dynamically weighs the importance of different time steps and variables, thereby improving feature representation. The standard scaled dot-product attention mechanism is defined as follows:

(8) $$\mathrm{Attention}\left(Q,K,V\right)=\mathrm{softmax}\left(\frac{QK^{T}}{\sqrt{d_{k}}}\right)V$$

where

- *Q* (Query): Represents the current time step information.
- *K* (Key): Stores information about the entire input sequence.
- *V* (Value): Stores the feature representations of the input sequence.
- $d_{k}$ is the dimensionality of the key vectors.

The computation process involves the following:

1. Computing similarity: Performing the dot product $QK^{T}$.
2. Scaling: Dividing by $\sqrt{d_{k}}$ to prevent gradient explosion.
3. Softmax normalization: Generating attention weights.
4. Weighted sum: Computing the final attention output.

To enhance feature learning, the Transformer employs multi-head attention, allowing multiple independent attention heads to focus on different aspects of the input:

(9) $$\mathrm{MultiHead}\left(Q,K,V\right)=\mathrm{Concat}\left({\mathrm{head}}_{1},{\mathrm{head}}_{2},\ldots ,{\mathrm{head}}_{h}\right)W^{O}$$

where

- *h* is the number of attention heads.
- Each ${\mathrm{head}}_{i}$ represents an independent attention mechanism.
- $W^{O}$ is a learnable linear transformation matrix.

Advantages of multi-head attention:

- Enables the model to capture different levels of features, improving representation capability.
- Avoids local optima that may arise from a single attention head.

#### 3.3.3. Positional Encoding

Since the Transformer lacks inherent recurrence, it explicitly incorporates sequential information via positional encoding, defined as follows:

(10) $$PE_{\left(pos,2i\right)}=\sin\left(\frac{pos}{{10,000}^{2i/d_{model}}}\right)$$

(11) $$PE_{\left(pos,2i+1\right)}=\cos\left(\frac{pos}{{10,000}^{2i/d_{model}}}\right)$$

where

- pos is the position index.
- *i* is the dimension index.
- $d_{model}$ is the embedding dimension.

Functionality:

1. Ensures temporally close time steps have similar representations.
2. Enables the Transformer to recognize sequence order, improving time series modeling.

#### 3.3.4. Multi-Variable Transformer Architecture

**Figure 6:**
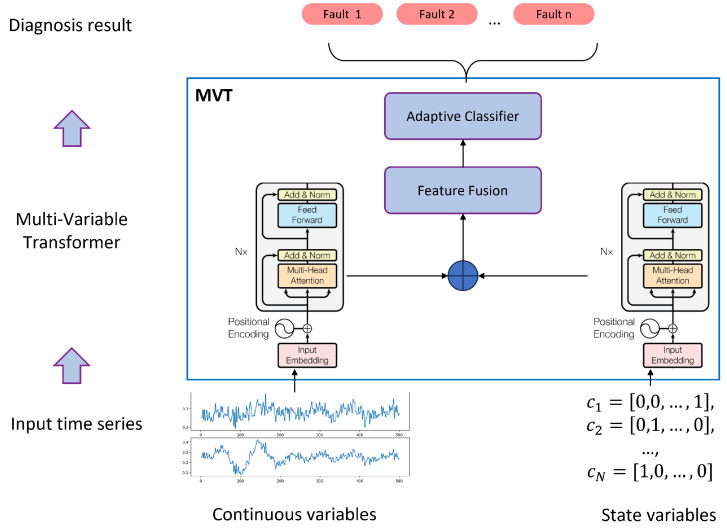
Multi-variable Transformer architecture.

Since fault diagnosis is a classification task, we use only the Transformer encoder without the decoder. Given that multi-variable data includes both numerical (analog) and categorical (state) data, we design two separate Transformer encoders for feature extraction. Figure 6 illustrates the multi-variable Transformer structure.

For analog variables, the embedding layer employs a fully connected Feed-Forward Network (FFN), whereas for categorical state variables, we utilize a Word2Vec-based embedding approach. Word2Vec maps discrete state variables into a continuous vector space, allowing them to be processed effectively by the Transformer encoder.

Finally, the outputs from both Transformer encoders are concatenated into a feature matrix (feature size M× sequence length *N*) and passed into an adaptive classifier to generate the final classification result.

### 3.4. Discussion on Time Complexity

During our research, we observed significant variations in computational time when using different types of neural networks as the backbone model for MAML, even with the same parameter scale. In fault diagnosis practices, if conditions permit, longer input sequences are often preferred to achieve higher diagnostic accuracy. Therefore, this section discusses the time complexity of MAML when LSTM, 1D CNN, and Transformer are used as the backbone models concerning the input sequence length *N*.

LSTM (Long Short-Term Memory) has a computational complexity of O(d) per time step, where *d* represents the hidden layer dimension. Therefore, for a sequence of length *N*, the total computational complexity of LSTM is O(Nd).

For a 1D Convolutional Neural Network (1D CNN), the computational complexity is primarily determined by the convolution operations. Assuming the input sequence length is *N*, the convolution kernel size is *k*, and the output dimension of the convolution layer is *d* (i.e., the number of channels), the computational complexity of the convolution operation is O(Nkd).

The computational complexity of a Transformer model is mainly attributed to the self-attention mechanism. The self-attention operation at each position involves pairwise interactions, leading to a complexity of $O(N^{2})$. Considering the feature dimension *d*, the computational complexity per layer is $O(N^{2}d)$, resulting in an overall complexity of $O(N^{2}d)$.

In MAML, gradient computation requires two forward passes and one backward pass. Therefore, the time complexity for different models combined with MAML is the following:

(12) O(Nkd)forCNN+MAML

(13) O(Nd)forLSTM+MAML

(14) $$O(N^{2}d)\;\mathrm{for}\;\mathrm{Transformer}\;+\;\mathrm{MAML}$$

From a theoretical perspective, the influence of sequence length *N* is most significant for the Transformer + MAML approach. This implies that the computational cost of MVMT increases with longer sequences. However, in practical experiments, we observed that when the sequence length is relatively short, the absolute training time of a Transformer with the same scale is comparable to that of LSTM, while CNN remains the fastest. As the sequence length increases, the computational burden of LSTM rises more sharply than that of the Transformer, whereas the computational time of the Transformer and CNN increases at a slower rate. This discrepancy between theoretical complexity and actual runtime can be attributed to GPU parallelism.

1. 1D CNN: Convolution operations exhibit high parallelism, especially on GPU, allowing for more efficient acceleration compared to LSTM and Transformer models. This advantage is particularly evident when processing long sequences and large batches.
2. LSTM: While LSTM can leverage GPU for batch computations, its inherently sequential nature limits parallel efficiency. Since each time step must be computed before the next one begins, LSTM computations remain fundamentally serial, resulting in a complexity of O(Nd) even on GPU.
3. Transformer: Owing to its fully parallelizable self-attention mechanism, Transformer models benefit significantly from GPU acceleration. Since attention computations at each position are independent, Transformers generally outperform LSTMs in terms of efficiency, particularly as sequence length *N* increases.

In conclusion, although Transformer models theoretically have higher computational complexity, GPU acceleration significantly mitigates their runtime cost in practice. Compared to LSTM, the Transformer becomes increasingly advantageous as the sequence length grows. Moreover, since MAML involves both inner-loop and outer-loop updates, the integration of the MAML algorithm further enhances the training efficiency of the Transformer model.

## 4. Experimental Setup

### 4.1. Datasets

We evaluate our approach on two datasets: Tennessee Eastman Process (TEP) dataset and Power-Supply System dataset.

#### 4.1.1. Dataset 1: TEP

**Figure 7:**
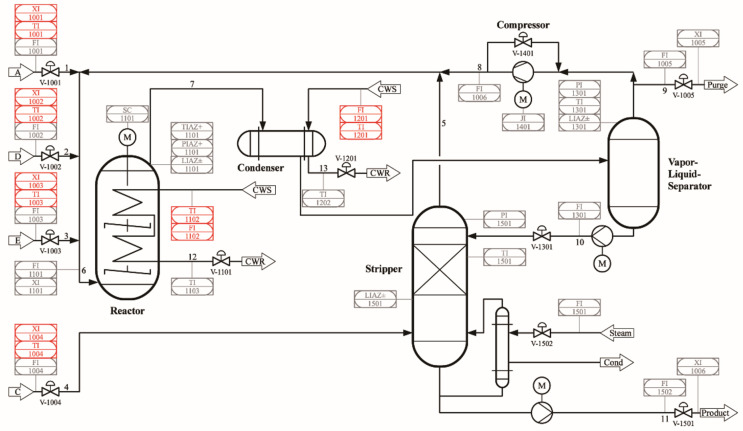
Tennessee Eastman process schematic diagram.

The Tennessee Eastman (TE) process is a well-established chemical process simulation system that represents a typical industrial chemical production process involving multiple unit operations and a set of adjustable variables. It simulates a large-scale chemical plant, with its core centered around an ethylene production process. Through precise modeling and simulation, the TE process captures common dynamic and nonlinear behaviors in chemical operations, including improper control actions, process faults, and other operational anomalies. It is widely utilized in research on process control, optimization, monitoring, and fault diagnosis. The TE dataset encompasses 20 typical fault modes observed in industrial processes, making it a standard benchmark for process monitoring and fault diagnosis studies. The process schematic diagram of TE process is shown in Figure 7. Further details can be found in Appendix A Table A1.

The TE process dataset consists of multiple subsets, with each fault mode containing both a training set and a testing set. The dataset size is as follows:

1. Training Set: The training set for each fault mode contains 480 samples.
2. Testing Set: The testing set for each fault mode contains 960 samples.

Each data sample contains 53 variables, including the following:

1. 40 Process Variables
2. 13 Manipulated Variables

A complete list of all variables is provided in Appendix A Table A2.

Considering that different fault types vary in detection difficulty, we allocated fault types with similar difficulty levels between the meta-training set and the meta-testing set based on diagnostic accuracy under sufficient sample conditions. The division of the fault types is shown in Table 1.

After dividing the fault types into meta-training and meta-testing sets, we construct two types of few-shot learning tasks:

- Task 1: Each task contains 5 fault types. In the meta-training set, each fault type includes **1** support sample and **5** query samples, whereas in the meta-testing set, each fault type includes **1** support sample and **30** query samples. The length of each sample is **50**.
- Task 2: Each task contains 5 fault types. In the meta-training set, each fault type includes **3** support samples and **10** query samples, whereas in the meta-testing set, each fault type includes **3** support samples and **30** query samples. The length of each sample is **30**.

The task construction details are summarized in Table 2.

#### 4.1.2. Dataset 2: Power-Supply System

This dataset comprises operational data from a real-world Power-Supply System of in-orbit spacecraft, including variables related to voltage, current, temperature, and other relevant parameters. It includes labeled instances of different fault conditions that occur in the system.

The dataset contains 24 types of faults, as listed in Appendix A Table A3.

Since the number of samples varies across different faults, we divide them into meta-training and meta-testing sets based on actual sample availability. The selected fault types for each set are listed in Table 3.

Based on the division of fault types, we construct three types of few-shot learning tasks:

- Task Setting 1: Each task in both the meta-training and meta-testing sets contains 5 fault types. The meta-training set includes **5** support samples and **10** query samples per task, while the meta-testing set includes **5** support samples and **30** query samples per task. The length of each sample is **30**.
- Task Setting 2: The meta-training set contains **5** fault types per task, whereas the meta-testing set contains **7** fault types per task. In the meta-training set, each task consists of **5** support samples and **10** query samples, while in the meta-testing set, each task consists of **5** support samples and **30** query samples. The length of each sample is **30**.
- Task Setting 3: Each task in both the meta-training and meta-testing sets contains 5 fault types. The meta-training set includes **3** support samples and **10** query samples per task, while the meta-testing set includes **3** support samples and **30** query samples per task. The length of each sample is **50**.

The details of task construction are summarized in Table 4.

### 4.2. Evaluation Metrics

The evaluation metrics are categorized into two dimensions: efficiency and performance.

For the efficiency dimension, we adopt the total meta-training time, denoted as $T_{\mathrm{training}}$, as the evaluation metric.

For the performance dimension, we measure the classification accuracy of the trained model (with optimal initialization) on the query samples of all tasks in the test set.

### 4.3. Hardware Platform

To ensure the reproducibility of the experiments, all experiments were conducted on the following hardware platform shown in Table 5.

### 4.4. Comparison Methods

To evaluate the effectiveness of the proposed method, we compare it against the following state-of-the-art meta-learning-based fault diagnosis approaches:

1. **LSTM-based meta-learning (meta-LSTM)** [18]
2. **1D CNN-based meta-learning (meta-CNN)** [19]
3. **ViT-based meta-learning (meta-ViT)** [20]

Furthermore, in Dataset 2, we incorporate the meta-SGD strategy to explore its impact on both efficiency and performance across different models.

### 4.5. Hyperparameter Settings

To ensure a rigorous and fair comparison, we conducted preliminary experiments using a sufficient number of samples from both Dataset 1 and Dataset 2. Each base model was optimized using Optuna, and the final tuned hyperparameters are presented in Table 6 and Table 7.

It is noteworthy that Dataset 2 contains both continuous (analog) and discrete (state) variables. Hence, the input feature size is configured to accommodate both data types.

## 5. Results and Discussion

### 5.1. Experimental Results on Dataset 1 (TEP)

Table 8 presents the results of training and testing directly on the meta-test task set without employing the MAML learning strategy. Here, MVT refers to Multi-Variable Transformer, which can handle multivariate time series but does not utilize the MAML learning method for training on the meta-test task set from scratch. Traditional deep learning models, such as 1DCNN and LSTM, perform relatively poorly, with accuracy below 40% in both settings, highlighting their limitations in handling small-sample, complex time series data. In contrast, Transformer-based models—ViT and the proposed MVT—demonstrate higher performance, with accuracy exceeding 43%. Notably, MVT achieves the highest accuracy in Setting 1 (44.15%), while ViT slightly outperforms it in Setting 2 (44.53%). These results indicate that, even without the MAML strategy, the proposed multi-variable Transformer architecture is effective in capturing complex temporal patterns, offering improved generalization over conventional models, especially in more challenging settings with only one support sample.

Table 9 summarizes the training time and classification accuracy of models incorporating the MAML strategy.

From the results, it is evident that the proposed MVMT model consistently achieves the highest accuracy across both task settings while requiring only half the training time of LSTM + MAML. Although 1DCNN + MAML exhibits the fastest training speed, significantly outperforming other methods in terms of efficiency, its final performance remains inferior to Transformer-based models and is comparable to LSTM. On the other hand, LSTM attains moderate accuracy but incurs the longest training time. Overall, these findings indicate that MVMT offers a clear and consistent advantage in few-shot fault diagnosis tasks for chemical processes.

Moreover, regardless of the model architecture, incorporating the MAML strategy enables the models to learn better initial parameters from the source domain, facilitating rapid adaptation to new tasks in the target domain.

### 5.2. Experimental Results on Dataset 2 (Power-Supply System)

Table 10 presents the experimental results on Dataset 2, where both analog and state variables are included. Under these conditions, MVMT demonstrates an even greater advantage.

In Task Setting 1 (5-way 5-shot classification), MVMT achieves the highest fault diagnosis accuracy while maintaining stable and efficient training performance. In contrast, LSTM requires the longest training time but fails to deliver accuracy improvements proportional to its computational cost. It is also noteworthy that the MAML algorithm consistently enhances each model’s adaptation to target-domain tasks, as parameters learned from the source domain significantly improve accuracy over random initialization.

When meta-SGD is introduced, the accuracy of 1DCNN and LSTM improves, while training time remains stable or even decreases slightly—likely due to hardware performance fluctuations. However, for Transformer-based models, the application of meta-SGD does not yield significant accuracy gains. In fact, for ViT, accuracy decreases from 78.40% to 72.66%, making it even less effective than LSTM+meta-SGD.

In Task Setting 2, the meta-test tasks involve 7-way classification to evaluate whether the adaptive classifier can handle varying class numbers. The results indicate that the classifier successfully adapts to this scenario, with MVMT again achieving the highest accuracy (73.00%). Although 1DCNN maintains a training time advantage, its accuracy drops significantly compared to Task Setting 1, suggesting that its combination with the adaptive classifier is less effective than MVMT. Regarding the impact of meta-SGD, there is a slight accuracy improvement for 1DCNN and ViT, while its effect on MVMT and LSTM is negligible. Additionally, in the baseline models without MAML, MVT achieves an accuracy close to that of MAML-enhanced 1DCNN, highlighting MVT’s inherent adaptability to different tasks.

Task Setting 3 involves longer sequences but fewer support samples (3-shot). Even under these conditions, MVMT remains the best-performing model, significantly outperforming others. When meta-SGD is introduced, 1DCNN and LSTM show notable accuracy improvements, whereas Transformer-based models see minimal gains. This observation suggests that Transformer models exhibit similar parameter update patterns during training, reducing the need for meta-SGD to adjust learning rates for individual parameters.

We can observe that MVMT demonstrates high and stable performance across two different datasets, highlighting the portability of the proposed method. This portability primarily arises from its Model-Agnostic Meta-Learning (MAML) foundation and the flexible Transformer architecture. MAML allows the model to learn generalizable initialization parameters, enabling rapid adaptation to new fault scenarios with limited data. Additionally, Transformers are inherently capable of handling variable-length, multivariate time series data without requiring task-specific customization. These characteristics make MVMT well suited for deployment in a wide range of large-scale systems.

## 6. Conclusions

This paper presents a novel approach to fault pattern recognition in complex engineering systems using meta-learning, with a specific focus on fault diagnosis and classification. The limitations of existing methods are analyzed, and improvements are made to the Transformer model. We introduce the Multi-Variable Transformer based on the Self-Attention Mechanism (MVMT), which combines the rapid adaptability of meta-learning with the powerful feature extraction capabilities of Transformer networks. MVMT effectively handles multi-variable fault pattern recognition, particularly in small-sample scenarios, demonstrating significant promise in both practical applications and theoretical advancements.

The key contributions of this paper are as follows:

- MVMT demonstrates exceptional feature extraction capabilities, particularly for time series data, and benefits from GPU-accelerated parallel computation, making it more computationally efficient compared to traditional RNNs and LSTMs.
- The model excels in rapidly adapting to new fault tasks with minimal data, addressing the challenge of fault categories with limited samples, which is crucial for complex systems.
- MVMT successfully integrates various types of variables (e.g., analog and status variables), offering high flexibility for deployment in diverse, multi-variable complex systems.
- The introduction of an adaptive classifier solves the issue of varying numbers of fault categories across tasks, overcoming a limitation in the original MAML approach.
- Comparative experiments conducted on the TE chemical process dataset and spacecraft telemetry dataset demonstrate that MVMT outperforms existing methods in multi-variable, small-sample fault diagnosis tasks for complex systems.

Looking forward, as the scale and complexity of engineering systems continue to grow, fault diagnosis tasks will face increasingly diverse and sophisticated challenges. One key direction for future research is to address real-time multi-fault pattern recognition in more complex systems. While traditional methods focus on detecting single fault modes, real-world applications often involve multiple concurrent fault modes or interactions between them. This necessitates the development of models capable of simultaneously recognizing and distinguishing multiple fault modes. Multi-Task Learning (MTL) methods, which optimize multiple fault diagnosis tasks concurrently, represent a promising avenue for enhancing model adaptability in such systems.

Another crucial area for future research is dealing with the increasing diversity of fault diagnosis data types, such as time series, image, and text data. Achieving effective cross-modal fusion and analysis will be vital for advancing fault diagnosis techniques. While Transformer models have shown success with multi-variable time series data, incorporating other data forms will require innovative approaches. For example, combining Graph Neural Networks (GNNs) with Transformer models could provide a better representation of global system information and the relationships between system components, improving fault diagnosis accuracy.

Lastly, as computational power continues to advance, applying fault diagnosis models in practical industrial environments—especially in smart manufacturing and the Industrial Internet of Things (IIoT)—will become an important research focus. In these applications, model interpretability is crucial, as industrial systems require a clear understanding of the decision-making process. Future research can focus on enhancing the transparency and explainability of deep learning models, aiming to develop fault diagnosis systems that are not only robust and efficient but also interpretable and understandable, meeting the needs of engineering practice.

By integrating and advancing these technologies, future complex engineering systems will be able to perform more intelligent fault detection and prediction, significantly enhancing system reliability and safety.

## Figures and Tables

**Table 1:** TEP dataset: fault types in meta-training and meta-testing sets.

Task Set	Selected Fault Types
Meta-Training Set	3, 5, 6, 7, 8, 9, 10, 11, 15, 16, 18, 19, 20
Meta-Testing Set	1, 2, 4, 12, 13, 14, 17

**Table 2:** Task settings for the TEP dataset.

Task Type	Dataset	Number of Fault Types	Support Samples	Query Samples	Sample Length
Task 1	Meta-Training Set	5	1	5	50
	Meta-Testing Set	5	1	30	50
Task 2	Meta-Training Set	5	**3**	**10**	**30**
	Meta-Testing Set	5	**3**	30	**30**

**Table 3:** Satellite data: fault types in meta-training and meta-testing sets.

Dataset	Selected Fault Types
Meta-Training Set	0, 1, 5, 8, 9, 11, 12, 14, 16, 17, 18, 19, 20, 22
Meta-Testing Set	2, 3, 4, 6, 7, 10, 13, 15, 21, 23

**Table 4:** Task settings for the satellite telemetry dataset.

Task Setting	Dataset	Number of Fault Types	Support Samples	Query Samples	Sample Length
Task Setting 1	Meta-Training Set	5	**5**	**10**	**30**
	Meta-Testing Set	5	**5**	**30**	**30**
Task Setting 2	Meta-Training Set	5	**5**	**10**	**30**
	Meta-Testing Set	**7**	**5**	**30**	**30**
Task Setting 3	Meta-Training Set	5	**3**	**10**	**50**
	Meta-Testing Set	5	**3**	**30**	**50**

**Table 5:** Experimental hardware configuration.

Hardware Component	Specification
CPU	AMD Ryzen 9 9800X3D
GPU	NVIDIA RTX 3090 24GB
RAM	48 GB DDR5
Storage	1 TB SSD
Operating System	Windows 11 Version 24H2

**Table 6:** Optimized hyperparameters for Dataset 1.

Model	Hidden Size	Feature Size	Attention Heads	Encoder Layers	Kernel Size
MVMT	128	256	3	2	–
LSTM	32	224	–	–	–
1DCNN	96	256	–	–	5
ViT	96	96	4	2	–

**Table 7:** Optimized hyperparameters for Dataset 2.

Model	Hidden Size	Feature Size	Attention Heads	Encoder Layers	Kernel Size
MVMT	256	256	8	4	–
LSTM	128	196	–	–	–
1DCNN	128	256	–	–	7
ViT	96	96	4	2	–

**Table 8:** Experimental results on Dataset 1: baseline models.

Task Setting Method	Setting 1 Accuracy	Setting 2 Accuracy
1DCNN	33.67%	38.00%
LSTM	30.14%	39.67%
ViT	43.55%	**44.53%**
**MVT (proposed)**	**44.15%**	43.76%

**Table 9:** Experimental results on Dataset 1: models with MAML strategy.

Task Setting	Setting 1		Setting 2	
Method	Accuracy	Training Time (s)	Accuracy	Training Time (s)
1DCNN + MAML	52.73%	**706**	60.94%	**903**
LSTM + MAML	50.05%	7749	61.33%	7670
ViT + MAML	53.47%	2826	63.80%	3172
**MVMT (proposed)**	**56.70%**	2867	**68.00%**	3165

**Table 10:** Experimental results on Dataset 2.

Task Setting	Setting 1		Setting 2		Setting 3	
Method	Accuracy	Training Time	Accuracy	Training Time	Accuracy	Training Time
1DCNN	16.30%	NaN	14.50%	NaN	19.52%	NaN
LSTM	31.40%	NaN	33.74%	NaN	32.86%	NaN
ViT	**36.73**%	NaN	26.67%	NaN	22.86%	NaN
**MVT (proposed)**	31.67%	NaN	**41.33**%	NaN	**33.64**%	NaN
1DCNN + MAML	64.45%	**1353**	43.63%	**1464**	65.53%	**1814**
LSTM + MAML	67.87%	5258	60.00%	5216	70.10%	8750
ViT + MAML	78.40%	2955	64.45%	2778	80.20%	3322
**MVMT (proposed)**	**83.20%**	2652	**72.10%**	2787	**80.86%**	3001
1DCNN + meta-SGD	68.9%	**1282**	47.92%	**1550**	69.87%	**1923**
LSTM + meta-SGD	73.34%	5250	60.00%	5640	79.05%	8917
ViT + meta-SGD	72.66%	2821	67.14%	2775	73.30%	3174
**MVMT + meta-SGD (proposed)**	**81.15%**	2542	**73.00%**	2918	**79.25%**	3111

## Data Availability

The data supporting the reported results are available at Github under the following link: https://github.com/YPCJ/MVMT.git (accessed on 20 March 2025).

## Appendix A. Dataset Detail

**Table A1:** Fault description in TEP dataset.

Fault	Description
IDV(1)	A/C Feed Ratio, B Composition Constant (Stream 4) Step
IDV(2)	B Composition, A/C Ratio Constant (Stream 4) Step
IDV(3)	D Feed Temperature (Stream 2) Step
IDV(4)	Reactor Cooling Water Inlet Temperature Step
IDV(5)	Condenser Cooling Water Inlet Temperature Step
IDV(6)	A Feed Loss (Stream 1) Step
IDV(7)	C Header Pressure Loss - Reduced Availability (Stream 4) Step
IDV(8)	A, B, C Feed Composition (Stream 4) Random Variation
IDV(9)	D Feed Temperature (Stream 2) Random Variation
IDV(10)	C Feed Temperature (Stream 4) Random Variation
IDV(11)	Reactor Cooling Water Inlet Temperature Random Variation
IDV(12)	Condenser Cooling Water Inlet Temperature Random Variation
IDV(13)	Reaction Kinetics Slow Drift
IDV(14)	Reactor Cooling Water Valve Sticking
IDV(15)	Condenser Cooling Water Valve Sticking
IDV(16)	Unknown
IDV(17)	Unknown
IDV(18)	Unknown
IDV(19)	Unknown
IDV(20)	Unknown

**Table A2:** Variables in TEP dataset.

Variable	Description	Variable	Description
XMV(1)	D Feed Flow (stream 2) (Corrected Order)	XMEAS(15)	Stripper Level
XMV(2)	E Feed Flow (stream 3) (Corrected Order)	XMEAS(16)	Stripper Pressure
XMV(3)	A Feed Flow (stream 1) (Corrected Order)	XMEAS(17)	Stripper Underflow (stream 11)
XMV(4)	A and C Feed Flow (stream 4)	XMEAS(18)	Stripper Temperature
XMV(5)	Compressor Recycle Valve	XMEAS(19)	Stripper Steam Flow
XMV(6)	Purge Valve (stream 9)	XMEAS(20)	Compressor Work
XMV(7)	Separator Pot Liquid Flow (stream 10)	XMEAS(21)	Reactor Cooling Water Outlet Temp
XMV(8)	Stripper Liquid Product Flow (stream 11)	XMEAS(22)	Separator Cooling Water Outlet Temp
XMV(9)	Stripper Steam Valve	XMEAS(23)	Component A
XMV(10)	Reactor Cooling Water Flow	XMEAS(24)	Component B
XMV(11)	Condenser Cooling Water Flow	XMEAS(25)	Component C
XMV(12)	Agitator Speed	XMEAS(26)	Component D
XMEAS(1)	A Feed (stream 1)	XMEAS(27)	Component E
XMEAS(2)	D Feed (stream 2)	XMEAS(28)	Component F
XMEAS(3)	E Feed (stream 3)	XMEAS(29)	Component A
XMEAS(4)	A and C Feed (stream 4)	XMEAS(30)	Component B
XMEAS(5)	Recycle Flow (stream 8)	XMEAS(31)	Component C
XMEAS(6)	Reactor Feed Rate (stream 6)	XMEAS(32)	Component D
XMEAS(7)	Reactor Pressure	XMEAS(33)	Component E
XMEAS(8)	Reactor Level	XMEAS(34)	Component F
XMEAS(9)	Reactor Temperature	XMEAS(35)	Component G
XMEAS(10)	Purge Rate (stream 9)	XMEAS(36)	Component H
XMEAS(11)	Product Sep Temp	XMEAS(37)	Component D
XMEAS(12)	Product Sep Level	XMEAS(38)	Component E
XMEAS(13)	Prod Sep Pressure	XMEAS(39)	Component F
XMEAS(14)	Prod Sep Underflow (stream 10)	XMEAS(40)	Component G
		XMEAS(41)	Component H

**Table A3:** Fault descriptions of Power-Supply System.

Number	Device	Unit	Fault Description
0	Power Controller	BCRB Module	Single Drive Transistor Open or Short in MEA Circuit
1	Power Controller	BCRB Module	Single Operational Amplifier Open or Short in MEA Circuit
2	Power Controller	BCRB Module	Incorrect Battery Charging Voltage Command
3	Power Controller	Power Lower Machine	CDM Module Current Telemetry Circuit Fault
4	Power Controller	Power Lower Machine	POWER Module Bus Current Measurement Circuit Fault
5	Power Controller	Power Lower Machine	Solar Array Current Measurement Circuit Fault
6	Power Controller	Power Lower Machine	Communication Fault
7	Power Controller	Global	Load Short Circuit
8	Power Controller	Power Module	S3R Circuit Diode Short Circuit
9	Power Controller	Power Module	S3R Circuit Shunt MOSFET Open Circuit
10	Power Controller	Power Module	Abnormal S3R Shunt Status
11	Power Controller	Distribution (Heater) Module	Y Board Heater Incorrectly On
12	Power Controller	Distribution (Heater) Module	Battery Heater Band Incorrectly Off
13	Power Controller	Distribution (Heater) Module	Battery Heater Band Incorrectly On
14	Solar Array	Isolation Diode	Short Circuit
15	Solar Array	Isolation Diode	Open Circuit
16	Solar Array	Interconnect Ribbon	Open Circuit
17	Solar Array	Bus Bar	Solder Joint Open Circuit
18	Solar Array	Solar Cell	Single Cell Short Circuit
19	Solar Array	Solar Cell	Single Cell Open Circuit
20	Solar Array	Solar Cell	Solar Cell Performance Degradation
21	Solar Array	Solar Wing	Single Subarray Open Circuit
22	Solar Array	Solar Wing	Single Wing Open Circuit
23	Solar Array	Solar Wing	Solar Wing Single Subarray Open Circuit
